# Advancing the analysis of water pipe failures: a probabilistic framework for identifying significant factors

**DOI:** 10.1038/s41598-024-69855-w

**Published:** 2024-08-19

**Authors:** Muhammad Muddassir, Tarek Zayed, Ridwan Taiwo, Mohamed El Amine Ben Seghier

**Affiliations:** 1 Department of Building and Real Estate, The Hong Kong Polytechnic University, 11 Yuk Choi Road, Hung Hom, Kowloon, Hong Kong China; 2 Department of Built Environment, Oslo Metropolitan University, Oslo, Norway

**Keywords:** Water distribution network, Reliability of WDNs, Failure factors of WDN, Water Pipes, Preventive maintenance, Bayes’ theorem, Probability of failure, Civil engineering, Hydrology

## Abstract

The failure of water pipes in Water Distribution Networks (WDNs) is associated with environmental, economic, and social consequences. It is essential to mitigate these failures by analyzing the historical data of WDNs. The extant literature regarding water pipe failure analysis is limited by the absence of a systematic selection of significant factors influencing water pipe failure and eliminating the bias associated with the frequency distribution of the historical data. Hence, this study presents a new framework to address the existing limitations. The framework consists of two algorithms for categorical and numerical factors influencing pipe failure. The algorithms are employed to check the relevance between the pipe’s failure and frequency distributions in order to select the most significant factors. The framework is applied to Hong Kong WDN, selecting 10 out of 21 as significant factors influencing water pipe failure. The likelihood feature method and Bayes’ theorem are applied to estimate failure probability due to the pipe materials and the factors. The results indicate that galvanized iron and polyethylene pipes are the most susceptible to failure in the WDN. The proposed framework enables decision-makers in the water infrastructure industry to effectively prioritize their networks’ most significant failure factors and allocate resources accordingly.

## Introduction

Water is an essential resource required for various human activities, like drinking, cooking, cleaning, and flushing, and it is transported through various types of water pipes. Water pipes often experience unprecedented failures associated with environmental, economic, and social consequences^1–3^. Regarding environmental concerns, water pipe failure leads to water loss, causing flooding and erosion. In 2014, a steel pipe with a diameter of 762 $mm$ in California, USA, experienced a failure resulting in multiple walkways and garages flooding. This incident caused extensive damage to hundreds of vehicles, as reported by^4^.

Similarly, the failure of water pipes can have significant economic consequences. In addition to repairing or replacing the failed pipes, there are indirect costs associated with water loss, property damage, and disruption of services^5^. For instance, it was reported that repairing and rehabilitating existing water distribution networks (WDNs) in the USA would cost over $1 trillion over the next few decades^6^. Additionally, Xu et al^7^ stated that China spent more than 10 billion RMB (or 1.45 billion USD) in 2014 to rehabilitate its WDNs. The failure of pipes poses a social concern as it increases health risks due to the potential contamination of water and the subsequent incidence of waterborne diseases. As per the study by^8^, the World Health Organization has estimated that globally, 3.41 million deaths occur annually due to water-related diseases.

Numerous factors influencing the failure of water pipes have been reported in the extant literature^9–11^. These factors have been broadly categorized into three: pipe-related, environment-related, and operational-related factors^12^. Pipe-related factors include age, material, diameter, length, manufacturing flaw, installation quality, and buried depth. Environment-related factors include corrosion, soil type, soil pH, atmospheric temperature, road traffic, soil composition and microbiological agents, and external loads. Similarly, internal pressure, water type, water hammering, and water quality are some of the operational-related factors influencing water pipe failure^13–17^. However, categorizing these factors is not always clear-cut, and some factors may overlap among different categories. For instance, corrosion can be influenced by both the environment and the material of the pipe. In addition, some factors may interact with each other, leading to complex and interdependent causes of pipe failure.

It is worth noting that the relative importance of the factors influencing pipe failure may vary depending on the specific context and location of the WDN. For instance^6^ found interval to last break, temperature changes, pipe length, and pipe age as the most significant factors leading to water pipe failure in the Cleveland WDN, USA. On the other hand, pipe material, length, age, and the number of previous failures were found to be the most influencing factors for a WDN located in Seville, Spain^18^. Therefore, a comprehensive and context-specific understanding of the potential failure factors is necessary to manage and maintain the WDN effectively.

As mentioned earlier, it is evidenced that water pipe failure is a severe problem that leads to several negative impacts. Hence, water pipe failure needs to be prevented as much as possible. One of the ways to mitigate the failure is to obtain the probability of failure of certain pipe material in the network given a particular factor and vice versa. For instance, the probability of cast iron (CI) pipe (i.e., material type) given its age can be obtained, and the probability that a pipe failed at a certain age given its material type is CI can be computed from the historical failure data of a WDN. These kinds of computations are referred to as conditional probabilities and can be derived using Bayes’ theorem. Singh^19^ and Tchórzewska-Cieślak et al.^20^ adopted Bayes’ theorem to determine the conditional failure probabilities of pipes in Honolulu’s WDN, USA, and Powiat city’s WDN, Poland, respectively. However, these studies are limited because the bias associated with the failure of historical data of a typical WDN was not addressed. The frequency distribution of the assets (i.e., pipe segments) in a WDN is often correlated with the frequency distribution of the failed assets^18^. For instance, if 80% of a network consists of ductile iron (DI) and the other 20% is made of several other pipe materials, there is a high possibility that a higher percentage of failure may be associated with DI in the network even if DI is not the most susceptible material. This kind of bias can be eliminated by investigating the frequency distribution of the total assets and that of the failed assets. Suppose a significant difference exists between the two distributions. In that case, the assets can be said to have failed due to the investigating factor and not because of their mere abundance in the network.

Zangenehmadar & Moselhi^21^ employed the Delphi method with the aid of mean ranking score analysis to prioritize factors influencing the failure of water pipes. The mean ranking score analysis depended on a questionnaire survey that was distributed among WDN experts. Furthermore, El Chanati et al.^22^, developed performance assessment models for water pipelines. These models were developed using data gathered from questionnaires distributed to water pipeline experts in Qatar. The weights of the factors were determined using four distinct methods: AHP, fuzzy AHP (FAHP), ANP, and fuzzy ANP (FANP). Similar qualitative approaches were adopted by Elshaboury et al.^23^ to rank the factors contributing to the failure of pipes in WDNs.

It is worth noting that the existing methods in the extant literature for identifying significant factors influencing the failure of pipes in WDNs are subjective in nature, and the results cannot be directly attributed to a specific WDN, as they are based on the general opinion of experts working in the field of WDNs. To fill the existing gaps in the literature, this study proposes a novel framework for analyzing the probability of failure of water pipes in a WDN. Specifically, the objectives of this study are as follows.

1. To develop a framework (for numerical and categorical data) for selecting the most significant factors affecting water pipe failure.
2. To quantify the reliability of WDN pipes (assets) in terms of probability computed from historical failure data while minimizing the abundance bias that commonly arises in calculating the failure probabilities using empirical methods.
3. To validate the proposed frameworks with the historical data of a real WDN.

This study has significant theoretical and practical implications. The proposed framework can advance the understanding of factors affecting water pipe failure in WDNs by providing a more systematic and data-driven approach. Given any material type, the framework can assist water utilities and municipalities in prioritizing their maintenance and replacement efforts by identifying the critical factors contributing to pipe failure. Hence, the contributions of this study to the field of WDNs can be summarized as follows:

• Identification of significant factors influencing water pipe failure
  The study introduces two new algorithms for selecting the most significant factors affecting water pipe failure. One algorithm is designed for numerical data, while the other is tailored for categorical data.
• Development of a new metric for failure probability
  A novel method for calculating failure probability is presented, accounting for the number of failures and the total length of pipes for each material. This approach provides a more balanced representation of failure risk, offering a more accurate vulnerability assessment across different pipe categories.
• Multi-factor analysis using conditional probability
  The study applies conditional probability to analyze multiple factors influencing pipe failures simultaneously. This comprehensive approach allows for a more nuanced understanding of how various factors interact to influence failure likelihood, moving beyond single-factor analyses common in previous studies.
• Application of the method to a complex, real-world WDN
  The methodology is applied to data from a large and diverse urban water network, demonstrating its applicability and effectiveness in real-world scenarios. This practical application provides relevant insights to practitioners, bridging the gap between theoretical models and utility management needs.

## Methodology

### Framework to find significant factors

Analyzing the failure probability of water pipes gives insight into the range of factors that could be associated with a high or low probability of failure. This method only relies on historical failure data of a network. For example, it is well-established that the probability of failure will be higher for the short-length pipes in a WDN and lower for longer pipes. Installation errors or ground movement can be the most influential reason for this behavior. A similar trend can be found in the diameter of pipes, i.e., a short diameter tends to have a higher failure probability than a larger diameter. The thicker wall thickness in large-diameter pipes and thinner in small-diameter pipes are primarily responsible for this trend.

Similar trends can be observed when examining additional parameters in any WDN. However, it is important to consider whether these trends are useful if they are consistent across all WDNs. While they may not provide definitive answers, they offer a probabilistic perspective by indicating which range of a specific factor is more likely to result in failures for a given material. However, these failure probability distributions are highly influenced by their frequency distributions. For example, the dataset analysis here shows a higher failure probability for shorter-length pipes, a well-reported fact in the literature^24,25^. However, the frequency distribution of the length of pipes shows that the WDN has a higher number of shorter-length pipes than pipes with longer lengths. This indicates that the failure distributions are highly influenced by their frequency distribution. Furthermore, the conclusions derived from all parameters’ failure distributions may not point to the root cause. In order to select the parameters whose failure distributions are not completely influenced by their frequency distribution, a generic framework is presented to test the significance of the parameters.

#### Numerical data

**Figure 1:**
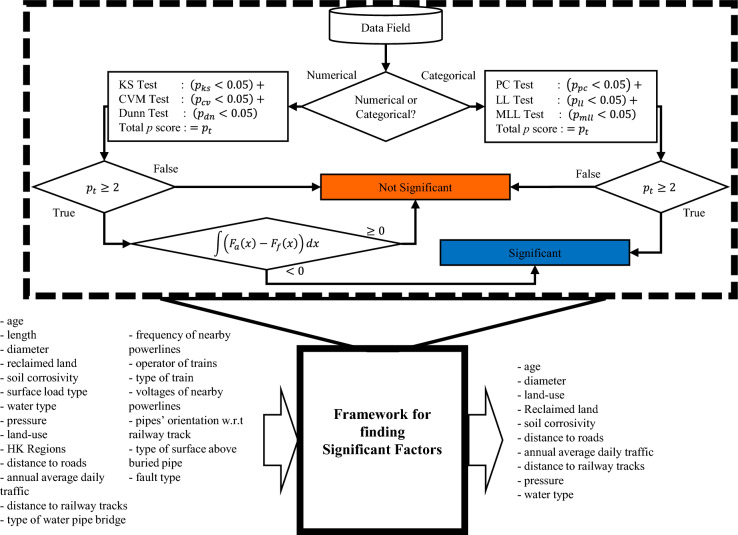
Generic Framework for Testing the Significance of a Parameter.

Figure 1 shows the flow diagram of the proposed algorithm. The algorithm can test the significance of numerical and categorical data based on their similarity or dissimilarity of cumulative failure distribution with their cumulative frequency distributions. Here, we compute the cumulative failure distribution from the data of failed pipes and the cumulative failure distribution from all pipes in the network (assets). In Fig. 1, the algorithm first determines the type of data present in the specified field. If the data is identified as numeric, the algorithm subsequently conducts three statistical tests to assess the null hypothesis ${(H}_{o})$. The three statistical tests are the Kolmogorov–Smirnov test ($ks$-test), Cramer-von Mises ($cv$-test), and Dunn’s test ($dn$-test) to check the goodness of fit of a cumulative failure distribution with its cumulative frequency distribution. The null hypothesis ${(H}_{o})$ in these tests, asset and failure datasets share the same cumulative probability distribution. Similarly, an alternative hypothesis $({H}_{a})$ states that the datasets of assets and failures are from different probability distributions. The null hypothesis is tested based on the $p$-value computed by each statistical test. If the $p$-value is smaller than $0.05$, the null hypothesis will be rejected, and the alternative hypothesis will hold. The null hypothesis will not be rejected if it equals or exceeds $0.05$. These tests’ cumulative scores are called the total $p$ score $\left({p}_{t}\right)$, as shown in Eq. (1)

1 $${p}_{t}=\left[{p}_{ks}<0.05\right]+\left[{p}_{cv}<0.05\right]+\left[{p}_{dn}<0.05\right]$$

where $\left[.\right]$ represents the Iverson brackets, which yields $1$ when the underlying condition is true and $0$ otherwise. ${p}_{ks}$, ${p}_{cv}$ and ${p}_{dn}$ denote the $p$-values from $ks$, $cv,$ and $dn$ statistical tests.

**Figure 2:**
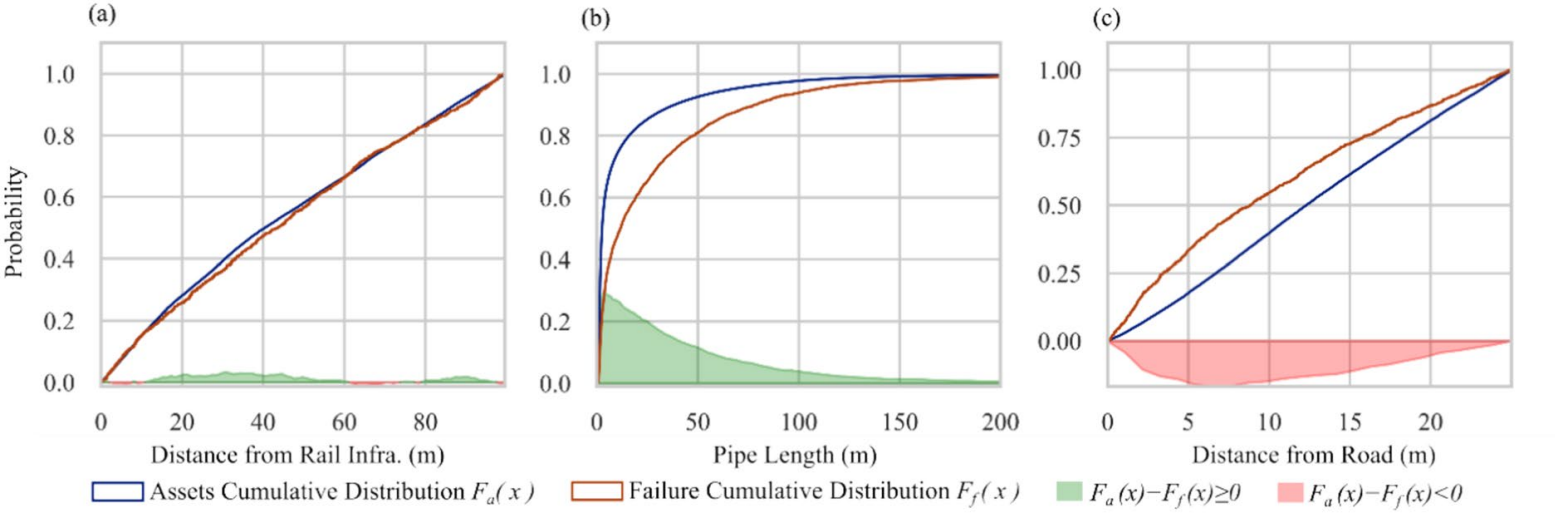
Three possible cases while comparing the cumulative distribution of assets and failure data. (**a**) If ${F}_{f}\left(x\right)$ closely follows the ${F}_{a}(x),$ the factor is considered insignificant, (**b**) If ${F}_{f}\left(x\right)$ reaches 1 slower than ${F}_{a}(x)$ , the factor is considered insignificant. (**c**) If ${F}_{f}\left(x\right)$ reaches 1 faster than ${F}_{a}(x)$ , the factor is deemed significant. These three graphs show real scenarios from our case study. (**a**) and (**b**) are a case of DI pipes where (**c**) is of S pipes.

To establish the significance of a factor, Fig. 1 shows that at least the $$p$$-values of two tests should satisfy the condition (i.e., $$<0.05).$$ The factor will be considered insignificant if $${p}_{t}$$ is smaller than $$2$$. Figure 2 shows three possible cases while comparing the cumulative distributions of assets and failure data. In the first case, both distributions closely follow each other, meaning cumulative failure distribution is highly influenced by its cumulative frequency distribution, as shown in Fig. 2(a). In this case, $${p}_{t}$$ would be less than 2, and the factor will be considered insignificant. In the second case, the cumulative distribution of assets and failure do not follow a similar trend. $${p}_{t}$$ would be equal to or greater than 2 in this case, as shown in Fig. 2(b). However, the cumulative failure distribution of failures is below the cumulative frequency distribution, $$\int \left({F}_{a}\left(x\right)-{F}_{f}\left(x\right)\right)dx\ge 0$$, showing that the tested factor is not significant. Figure 2(c) shows the case where $${p}_{t}$$ will be equal to or greater than 2 and $$\int \left({F}_{a}\left(x\right)-{F}_{f}\left(x\right)\right)dx<0$$. Here, the tested factor will be considered significant if the cumulative failure distribution shows a higher failure over the range of a factor than its cumulative frequency distribution. The empirical rationale behind this hypothesis is that the probability density distribution of failure data is narrower than that of frequency (assets) data when the number of failures in a specific range of a field is not proportionate to other ranges (proportionate according to failure density distribution). This situation leads to a steeper failure cumulative distribution than its frequency cumulative distribution, as shown in Fig. 2.

#### Categorical data

Three statistical significance tests are employed to find the significant categorical fields. For the categorical data, Pearson Chi Test ($pc$-test), log likelihood ($ll$-test), and modified log likelihood ($pmll$- test) tests are used to test the relevance of assets and failure data. The total score ${p}_{t}$ from the three statistical tests are denoted in Eq. (2). To determine the significance of a factor, Fig. 1 demonstrates that the p-values of at least two tests should meet the condition of being less than 0.05.

2 $${p}_{t}=\left[{p}_{pc}<0.05\right]+\left[{p}_{ll}<0.05\right]+\left[{p}_{mll}<0.05\right]$$

where $\left[.\right]$ represents the Iverson bracket, which equals $1$ when the underlying condition is true and equals $0$ otherwise. ${p}_{pc}$, ${p}_{ll}$ and ${p}_{mll}$ denote the $p$-values from $pc$-, $ll\ge$-, and $mll$- statistical tests.

### Probability of failure

The framework explained in “Framework to find significant factors” selects the most significant factors relating to the historical data of the WDN. After selecting the most significant factors of various materials, the probability of failure is computed for these factors. The probability of failure quantifies the risk of breakage associated with a specific pipe material. These probabilities can be computed from the historical failure data from water supply companies.

#### Conventional method

Conventionally, the probability of the failure of a certain material pipe is calculated by counting the number of failures in that material pipe divided by the total number of failures in the whole WDN.

3 $$P\left({M}_{i}\right)=\frac{{N}_{i}}{{N}_{T}}$$

$P\left({M}_{i}\right)$ is the probability of a pipe failure occurring in material i, calculated using the conventional method, ${N}_{i}$ denotes the total number of failures in $i$-th material and ${N}_{T}$ the total number of failures among other material pipes in the WDN. This method of computing the probability of failures from historical failure data indicates that each failure in the WDN has an equal likelihood of failure. While this method of calculating failure probabilities offers a broad perspective on the condition of a WDN, it is important to acknowledge that this assumption may oversimplify the complexities and interdependencies involved in computing failure probabilities within a WDN.

The total number of assets (pipe segments) in the water pipeline in Hong Kong is around 1.1 million. The number of failures observed in these assets in the last eleven years (2010–2020) is merely 40 thousand, representing about 4% of total WDN assets. Thus, the relative frequency method in Eq. (3) cannot provide a realistic estimate of failure probabilities for different material pipes. The total number of failures is much lower than the total assets. This means that the probability values from Eq. (3) cannot justify the failure probabilities of 1.1 million assets from only $4\text{\%}$ of failures. This implies that the failure probabilities need to be inferred from a smaller number of failures (as compared to the total number of assets in the WDN).

#### Likelihood feature method

The common feature method is proposed to tackle the problem of computing failure probability from the lower percentage of failure out of the total assets. This method incorporates assigned weights instead of counting the pipe failures. The probability of failure with the empirical method can be expressed as:

4 $$P\left({M}_{i}\right)=\frac{{\Sigma }_{j}{w}_{ij}}{{\Sigma }_{k}{w}_{k}}$$

where ${w}_{i,j}$ is the weight associated with $j$-th failed asset of $i$-th material, and ${w}_{k}$ is the weight of $k$-th failed asset, including all the other material pipes in a WDN. ${w}_{i,j}$ is computed by averaging the failure rate (failure/km/year) for the last 11 years for all materials and assigned this averaged failure rate to each pipe based on its length.

5 $${w}_{i,j}={f}_{i}\times {l}_{ij}$$

Here, ${f}_{i}$ denotes the average failure rate of $i$-th material and ${l}_{ij}$ the length of the pipe $i$-th material and $j$-th failed material.

**Figure 3:**
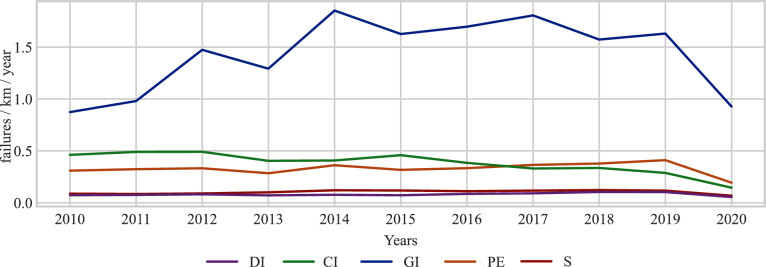
Failure rates among different pipe materials in the last eleven years.

Figure 3 shows the failure rate of different pipe materials found in the WDN. In Fig. 3, the failure rate is expressed as the number of failures per kilometer per year. The failure rates provide a rough estimate of the reliability and performance of different material types in a WDN. Also, it identifies the type of pipes where improvements in repair or replacement may be necessary to improve the overall performance of the WDN. We propose utilizing the sum of failure rates for different material types as a weight $({w}_{i,j})$, as depicted in Eq. (5). The rationale behind this approach is that higher failure rates indicate a greater probability of failure for a specific material type. Conversely, in the event of a failure within the WDN, it is reasonable to anticipate a higher likelihood of failure in the pipe material with a higher failure rate. The failure rate in a WDN (failures/km/year) allows for an accurate assessment of the network’s condition by considering failures relative to the network length. Analyzing failure data per kilometer per year helps identify vulnerable sections, prioritize maintenance, and optimize renewal strategies based on failure patterns^27–29^. Another metric for failure rate found in the literature is failures/year/km. The difference lies in their interpretation: failures/year/kilometer is more suitable for a spatial context, while failures/kilometer/year is more intuitive from a temporal perspective. The dataset provided by the WSD is historical failure data of the WDN, and failures/kilometer/year appear to be a better candidate for assessing the reliability of different pipe materials in the temporal context.

**Figure 4:**
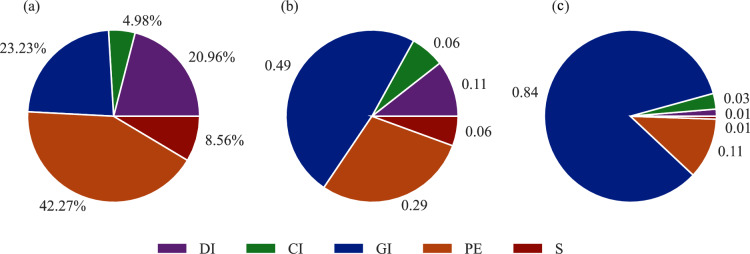
(**a**) Pie chart of the constituent of HK’s WDN; all constituent portion is shown in percentage. Pie Chart of the probability of failure of each material pipe in the WDN using the (**b**) common feature method and (**c**) the proposed method.

Figure 4(a) presents the material distribution within the network, representing the percentages of each material. Meanwhile, Fig. 4(b) and (c) display pie charts illustrating the probability of failures in the Water Distribution Network (WDN) using conventional and proposed methods, respectively. In Fig. 4(b), it is observed that pipes made of galvanized iron (GI) are more prone to failures compared to pipes of other materials, while the probability of failure for cast iron (CI) and steel (S) pipes is relatively lower. However, Fig. 4(c) reveals a significant difference, showing that CI pipes may experience three times more failures than S pipes. This disparity in failure probabilities between CI and S pipes results from the proposed method, which incorporates historical data and failure rates. The higher failure rate experienced by GI pipes contributes to their elevated failure probability, as determined by Eq. (4).

### Conditional probability of failure

Calculating failure probabilities based on historical data emphasizes the extent of failures in specific pipe materials within the network. However, it offers only a surface-level understanding of failures across different materials. Considering that the number of pipes in the WDN can impact the failure probabilities is justifiable. In other words, if the failure of pipes follows a uniform univariate random process, it is likely that the greater the number of pipes made of a particular material in the WDN, the higher the likelihood of failure for that material. However, water pipeline failures are highly complicated and multivariate random processes. Moreover, deducing or concluding from the univariate failure probability analysis could not suffice for an informed decision.

The failure probabilities obtained from historical data using Eq. (4) do not account for the influence of intrinsic, extrinsic, or operational factors of the pipes. To address this limitation, conditional probability offers a means to redistribute the likelihood of failure in pipes made of a specific material, considering various factors. In this context, conditional probability can be interpreted as a normalized failure probability relative to the total failure probability of pipes belonging to a particular material type, as demonstrated in Eq. (6).

6 $$P\left({F}_{l}|{M}_{i}\right)=\frac{P\left({F}_{l}\cap {M}_{i}\right)}{P\left({M}_{i}\right)}$$

where $P\left({F}_{l}|{M}_{i}\right)$ is the conditional probability of failure due to $l$-th factor given the pipe of $i$-th material, and $P({F}_{l}\cap {M}_{i})$ is the joint probability of failure due $l$-th factor and $i$-th material type.

Using conditional probability enables the evaluation of failure probability for a specific factor range, considering the material of the pipes within the WDN. This approach provides a normalized metric for analyzing pipe failures, allowing for comparisons across different materials on a consistent scale ranging from 0 to 1. By employing this normalized metric, it becomes possible to analyze Polyethylene (PE) and Cast Iron (CI) pipes on an equal scale, regardless of their varying numbers within the WDN. Figure 4(a) illustrates this comparison, where PE pipes constitute approximately 42% of the WDN while CI pipes comprise only around 5%.

### Case study

The pipeline data analyzed in this study is provided by the Water Supply Department (WSD) of Hong Kong. WSD is a governmental department responsible for managing, installing, and repairing water infrastructure, including water reservoirs, pumping stations, and water pipelines in Hong Kong. Hong Kong, positioned in the southern part of China, is a coastal city that shares its borders with Guangdong Province. It is situated at the mouth of the Pearl River Delta, south of Shenzhen. Geographically, Hong Kong is divided into three main regions: Hong Kong Island, Kowloon, and the New Territories. The city has a hilly and mountainous terrain with steep slopes and lowlands in the northern part of Hong Kong. It is famous for its highest population density, around 6300 persons per square kilometer. This encourages the governments to reclaim the land area for residential and economic zones. Around 70 km-square of land in Hong Kong is sea-reclaimed, about 7% of Hong Kong’s surface area (Development Bureau and Construction Industry^30^.

**Figure 5:**
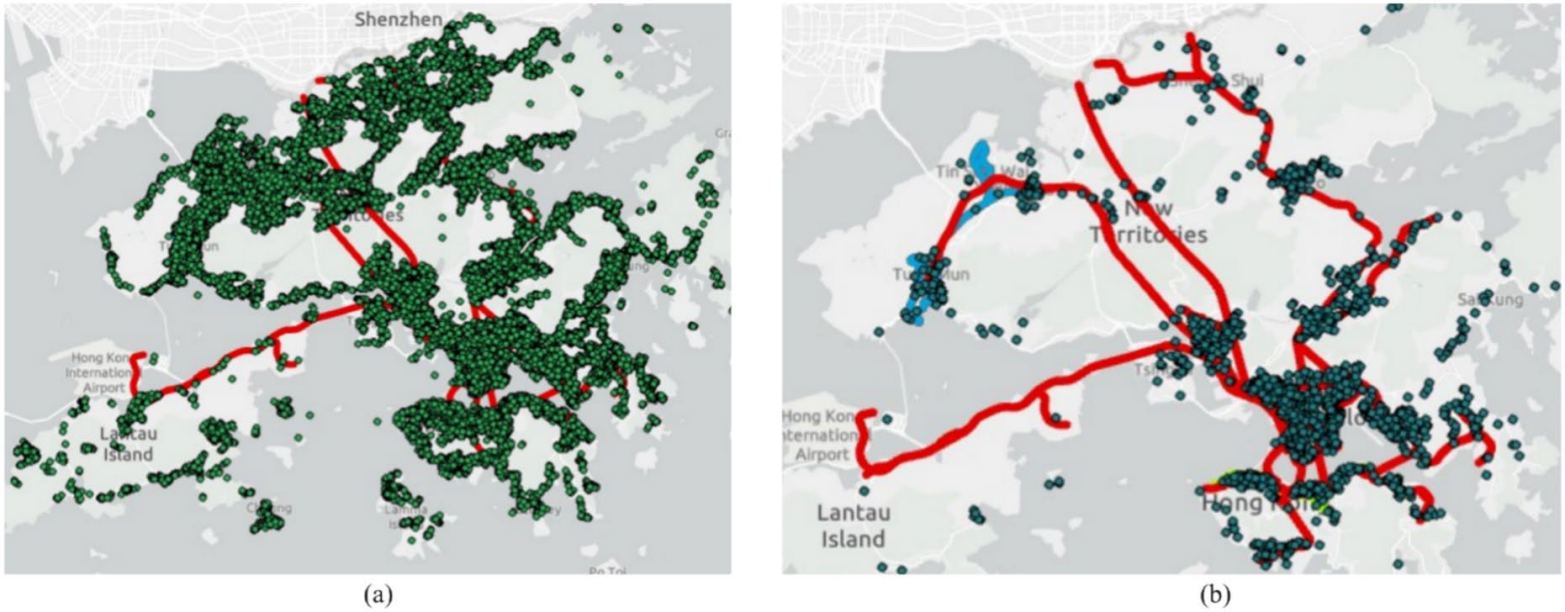
Geographical locations of failures in HK WDN from 2010–2020. (**a**) Leaks. (**b**) Bursts. The red line denotes the Mass Transit Railway (MTR), and the blue line represents the light rail line. This map was generated from the data provided by WSD HK using ArcGIS Pro 3.0 accesible at Esri official website (Esri^26^).

The data obtained from HK WSD encompasses a comprehensive 11-year record (2010–2020) of water pipe failures, including leaks and bursts, as displayed in Fig. 5. A leak is characterized by a smaller, often undetected water loss that may not immediately disrupt service, typically occurring at joints, fittings, or through small cracks. Leaks can persist for extended periods and are often identified during routine inspections. In contrast, a burst is a more severe failure involving a significant and sudden water loss, usually resulting from a complete fracture or large rupture in the pipe. Bursts often lead to immediate service disruptions and require urgent repair^31–33^.

The WSD database presents this information in a tabular format, capturing intrinsic and extrinsic parameters of each asset (pipe segment), along with details on the type, cause, and nature of the failures. The WDN in Hong Kong spans a total length of approximately 8000 km. Within this network, around 80% is dedicated to distributing fresh water, while the remaining 20% serves as conduits for saltwater supply to consumers. The primary constituents of Hong Kong’s WDN consist of pipes made from polyethylene (PE), galvanized iron (GI), and ductile iron (DI), accounting for approximately 42%, 23%, and 21%, respectively. The remaining 11% comprises steel (S), cast iron (CI), and other types of pipes, as depicted in Fig. 4(a).

## Result and discussion

HK’s WDN comprises about 1.1 million pipe segments, treated as individual pipe segments or assets in this study based on the proprietary data from WSD HK. The water supply department (WSD) provides the data associated with these pipes. The data provides information about the network, which is grouped into pipe-related, environment-related, and operation-related factors. Data cleaning was done to select relevant columns out of the 150 columns in the raw data.

We have selected the fields of data that contain the pipe age, diameter, and length under the pipe-related category; relevant fields that lie under environment-related factors include reclaimed land, soil resistivity, surface load type, land use, city regions, annual average daily traffic (AADT), distance from the road and distance from the rail infrastructure, amongst others. The internal water pressure on each pipe and the type of water have been selected as the operation-related factors (see Fig. 1).

Although the WDN consists of 14 types of pipe material, the network’s top five most abundant materials have been selected for brevity’s sake. The selected materials include cast iron (CI), ductile iron (DI), galvanized iron (GI), polyethylene (PE), and steel (S). The framework presented in “Framework to find significant factors” was applied to analyze 21 data fields across 5 pipe types. This analysis, illustrated in Fig. 1, aimed to identify the most significant factors influencing pipe failure for each material. Applying the algorithm proposed in “Methodology” revealed that only 10 of the 21 factors have a significant impact. These key factors are age, diameter, land use, soil corrosivity, reclaimed area, distance from the road, traffic, distance from the mass transit railway, internal water pressure, and water type. These findings provide a focused set of variables for further analysis and modeling of pipe failure probabilities in water distribution networks.

**Figure 6:**
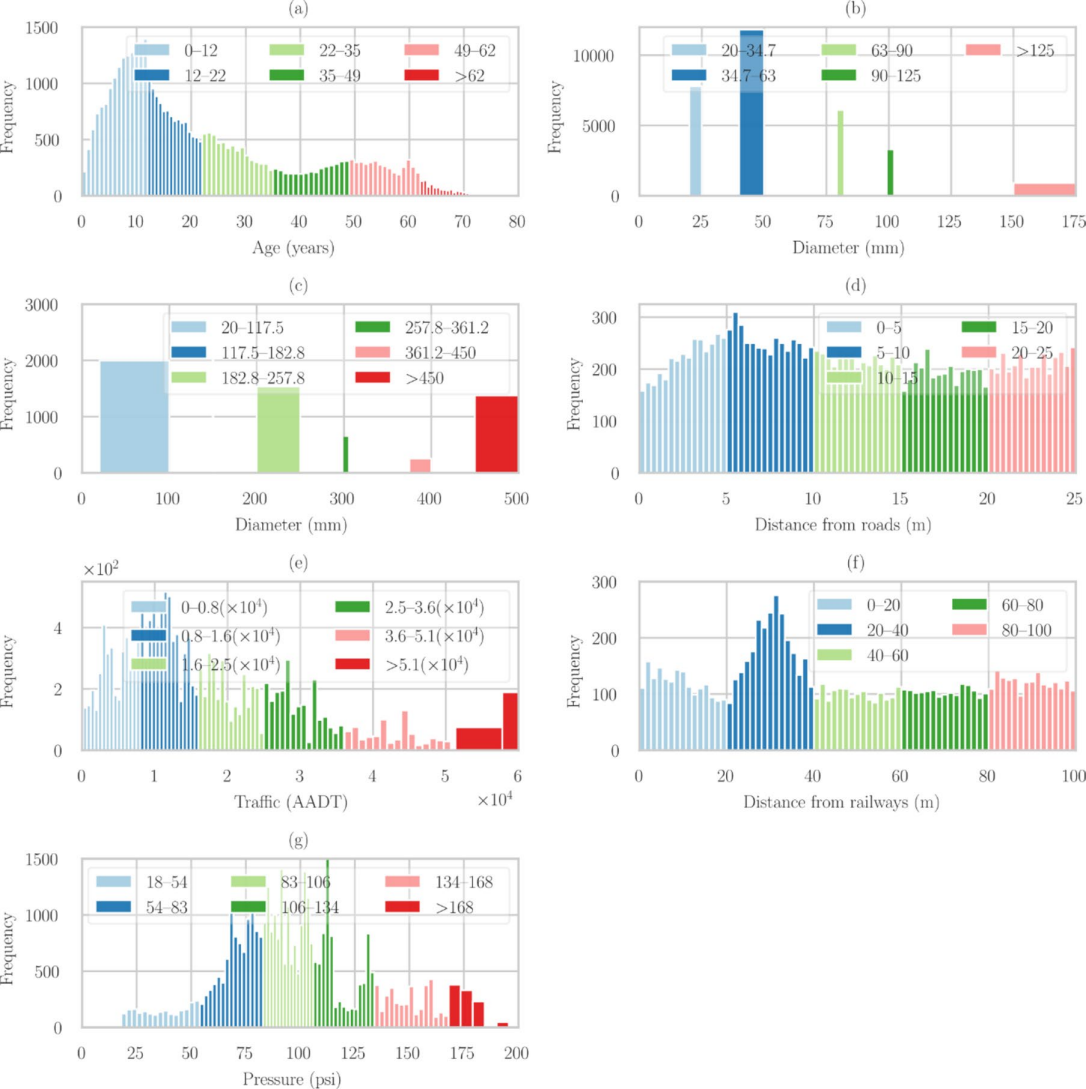
Automated clustering of numerical data using K-Means binning. (**a**) age, (**b**) diameter of Group A, (**c**) diameter of Group B, (**d**) distance from the roads, (**e**) annual average daily traffic, (**g**) distance from the mass transit railway system, and (**g**) operating pressure.

Numerical data was transformed into ordinal data using a binning approach to facilitate the computation of failure probabilities. The K-means algorithm was employed to determine optimal bin ranges, resulting in variable bin lengths tailored to the data distribution^34,35^. This automated clustering method ensures that the discretization process captures meaningful patterns in the continuous variables. Figure 6 illustrates the outcomes of this K-means-based clustering for various numerical factors, including age, diameter, traffic (AADT), distance from roads and railways, and pressure. Each subplot demonstrates how the continuous data has been effectively categorized into distinct, data-driven bins, providing a foundation for subsequent probability calculations and analysis. K-means binning strategy ensures automatic binning of numerical data and forms the bins based on the data spread. This feature enables automatic binning of highly sparse data, e.g., water pipes available in the market are discrete diameters, making the diameter data sparse.

To convert numerical data into categorical data using the K-means algorithm, one begins by choosing the number of clusters $k$, and initializing $k$ centroids (here, $k$ was set to 6). Each data point ${x}_{i}$ is then assigned to the nearest centroid using the Euclidean distance, defined as ${c}_{i}= \text{arg mi}{\text{n}}_{j} {\left| {x}_{i} - {\mu }_{j} \right|}^{2}$. Subsequently, centroids are recalculated as the mean of all points in each cluster, ${\mu }_{j} =\frac{1}{|{C}_{j}|} {\sum }_{{x}_{i}\in {C}_{j}}{x}_{i}$. This assignment and update process is repeated until convergence. Finally, each data point is assigned to a cluster, which can then be labeled with categorical labels, thereby transforming numerical data into categorical data.

Further, the probability of failures of water pipes due to a factor given its material is computed using Eq. (6) and presented in tabular forms in the subsequent sections. The last column of Tables1, 2, 3, 4, 5, 6, 7, 8, 9, 10, 11, 12, 13, 14, 15, 16, 17, 18, 19, 20, 21,22 represents the cumulative length of each row.

**Table 1:** Probability of failure of pipe of age (years) given materials $P\left({F}_{l}|{M}_{i}\right)$.

	0–12	12–22	22–35	35–49	49–62	> 62	${l}_{r}$
CI	0.007 (6.78)	0 (2.63)	0.017 (0.23)	0.262 (5.65)	0.537 (33.29)	0.176 (51.41)	515
PE	0.456 (56.79)	0.328 (34.14)	0.123 (4.62)	0.054 (0.94)	0.034 (1.48)	0.006 (2.02)	2835
DI	0.165 (13.61)	0.367 (39.81)	0.431 (40.77)	0.034 (5.34)	0.004 (0.05)	0 (0.43)	3211
S	0.135 (12.61)	0.283 (12.8)	0.33 (16.76)	0.147 (11.75)	0.091 (31.06)	0.013 (15.01)	2199
GI	0.24 (17.15)	0.338 (40.49)	0.205 (21.18)	0.1 (6.88)	0.096 (9.79)	0.022 (4.52)	1127

**Table 2:** Probability of failure of pipe materials given pipe age (years) $P\left({M}_{i}|{F}_{l}\right)$.

	CI	PE	DI	S	GI	${l}_{r}$
0–12	0 (1.37)	0.395 (63.08)	0.043 (17.11)	0.012 (10.86)	0.548 (7.57)	2553
12–22	0 (0.45)	0.241 (32.29)	0.082 (42.65)	0.022 (9.39)	0.655 (15.22)	2998
22–35	0.002 (0.06)	0.148 (6.4)	0.157 (63.9)	0.042 (17.99)	0.651 (11.65)	2049
35–49	0.053 (5.17)	0.148 (4.73)	0.028 (30.43)	0.043 (45.89)	0.727 (13.77)	563
49–62	0.117 (17.01)	0.102 (4.17)	0.003 (0.15)	0.028 (67.74)	0.75 (10.94)	1008
> 62	0.163 (36.94)	0.073 (7.99)	0 (1.92)	0.018 (46.04)	0.746 (7.1)	717

**Table 3:** Probability of failure of Group A pipes with certain diameters pipes (mm) given materials $P({F}_{l} |{M}_{i} )$.

	20–34.74	34.74–63	63–90	90–125	> 150	${l}_{r}$
PE	0.045 (5.45)	0.325 (22.81)	0.256 (25.21)	0.263 (19.95)	0.111 (26.57)	2829
GI	0.144 (17.37)	0.43 (39.66)	0.319 (31.25)	0.092 (9.85)	0.015 (1.88)	1043

**Table 4:** Probability of failure of Group A of pipes with certain material given various diameters pipes (mm) $P({M}_{i} |{F}_{l} )$.

	PE	GI	${l}_{r}$
20–34.7	0.115 (46)	0.885 (54)	335
34.7–63	0.236 (60.95)	0.764 (39.05)	1059
63–90	0.248 (68.64)	0.752 (31.36)	1039
90–125	0.539 (84.61)	0.461 (15.39)	667
> 150	0.746 (97.46)	0.254 (2.54)	771

**Table 5:** Probability of failure of Group B pipes with certain diameters pipes (mm) given materials $P({F}_{l} |{M}_{i} )$.

	20–117.45	117.4–182.8	182.8–257.8	257.8–361.2	361.2–450	> 450	${l}_{r}$
CI	0.222 (15.6)	0.354 (37.27)	0.215 (19.91)	0.082 (11.3)	0.067 (6.64)	0.061 (9.28)	515
DI	0.171 (12.54)	0.304 (25.2)	0.284 (22.96)	0.112 (17.19)	0.037 (6.35)	0.093 (15.76)	3293
S	0.043 (3.05)	0.076 (3.1)	0.063 (3.06)	0.048 (7.06)	0.028 (1.03)	0.742 (82.71)	2195

**Table 6:** Probability of failure of Group B pipes with certain material given various diameters pipes (mm) $P({M}_{i} |{F}_{l} )$.

	CI	DI	S	${l}_{r}$
20–117.5	0.226 (14.34)	0.71 (73.72)	0.064 (11.94)	560
117.5–182.8	0.208 (17.62)	0.727 (76.15)	0.065 (6.24)	1090
182.8–257.8	0.146 (11.08)	0.791 (81.66)	0.063 (7.26)	926
257.8–361.2	0.135 (7.47)	0.75 (72.65)	0.114 (19.88)	779
361.2–450	0.258 (12.87)	0.584 (78.66)	0.157 (8.47)	266
> 450	0.04 (2.01)	0.249 (21.79)	0.711 (76.21)	2382

**Table 7:** Probability of failure of pipes in specific land use given pipe material $P\left({F}_{l}|{M}_{i}\right)$.

	URBAN	RURAL	WATERBODY	SEA	${l}_{r}$
CI	0.969(95.87)	0.03(4.12)	0(0.01)	0(0.01)	491
PE	0.689(58.48)	0.31(39.55)	0(0.06)	0(1.9)	2843
DI	0.853(82.54)	0.145(17.1)	0.001(0.07)	0.001(0.3)	3293
S	0.826(42.27)	0.159(11.12)	0.003(0.15)	0.012(46.47)	2200
GI	0.424(41.18)	0.57(58.34)	0.004(0.27)	0.002(0.2)	1117

**Table 8:** Probability of failure in pipe material given land use $P\left({M}_{i}|{F}_{l}\right)$.

	CI	PE	DI	S	GI	${l}_{r}$
URBAN	0.031 (7.54)	0.314 (26.64)	0.117 (43.55)	0.041 (14.9)	0.498 (7.37)	6241
RURAL	0.001 (0.78)	0.169( 43.18)	0.024 (21.62)	0.009 (9.39)	0.797 (25.03)	2604
WATERBODY	0.003 (0.39)	0.004 (16.09)	0.021 (22.25)	0.037 (31.79)	0.934 (29.47)	10
SEA	0 (0)	0.031 (4.97)	0.05 (0.89)	0.179 (93.93)	0.74 (0.2)	1088

**Table 9:** The probability of failure of pipe in the soil of various corrosion levels (LPR) given pipe material $P\left({F}_{l}|{M}_{i}\right)$.

	HC	MC	NC	${l}_{r}$
CI	0.064(8.74)	0.694(69.53)	0.242(21.73)	491
PE	0.135(11.56)	0.743(76.89)	0.122(11.55)	2843
DI	0.138(11.66)	0.69(76.24)	0.171(12.09)	3293
S	0.16(32.07)	0.7(60.1)	0.141(7.83)	2200
GI	0.171(18.31)	0.764(75.44)	0.065(6.25)	1117

Here, HC denotes the highly corrosive, MC the mildly corrosive, and NC the non-corrosive soil-type defined by WSD HK.

**Table 10:** The probability of failure in pipe material given the soil of various corrosion levels (LPR) $P\left({M}_{i}|{F}_{l}\right)$.

	CI	PE	DI	S	GI	${l}_{r}$
HC	0.007 (2.58)	0.21 (19.73)	0.065 (23.05)	0.027 (42.36)	0.69 (12.28)	1666
MC	0.016 (4.74)	0.244 (30.35)	0.069 (34.86)	0.025 (18.35)	0.647 (11.7)	7203
NC	0.045 (9.92)	0.329 (30.53)	0.139 (37.04)	0.041 (16.02)	0.446 (6.5)	1075

Here, HC denotes the highly corrosive, MC the mildly corrosive, and NC the non-corrosive soil-type defined by WSD HK.

**Table 11:** The probability of failure of pipes in reclaimed land given pipe materials. Y means pipe in reclaimed land, and N means pipes in normal land $P\left({F}_{l}|{M}_{i}\right)$.

	N	Y	${l}_{r}$
CI	0.717(70.64)	0.283 (29.36)	489
PE	0.788(81.86)	0.212 (18.14)	2785
DI	0.679(74.5)	0.321(25.5)	3266
S	0.783(80.08)	0.217(19.92)	1233
GI	0.919(90.84)	0.081(9.16)	1103

**Table 12:** The probability of failure of pipe materials given reclaimed land.

	**CI**	**PE**	**DI**	**S**	**GI**	${{\varvec{l}}}_{{\varvec{r}}}$
N	0.014 (4.9)	0.227 (32.35)	0.059 (34.53)	0.024 (14.01)	0.675 (14.22)	7047
Y	0.035 (7.85)	0.378 (27.63)	0.174 (45.56)	0.042 (13.43)	0.371 (5.53)	1828

Y means pipe in reclaimed land, and N means pipes in normal land $P\left({M}_{i}|{F}_{l}\right)$.

**Table 13:** The probability of failure of pipes within 25 m of road given pipe materials $P\left({F}_{l}|{M}_{i}\right)$.

	0–5	5–10	10–15	15–20	20–25	${l}_{r}$
CI	0.194 (20.95)	0.1 (12.39)	0.063 (7.51)	0.07 (9.35)	0.572 (49.79)	372
PE	0.327 (28.34)	0.167 (17.12)	0.105 (13.91)	0.116 (14.2)	0.284 (26.43)	1089
DI	0.27 (23.79)	0.136 (15.16)	0.061 (8.42)	0.081 (10.69)	0.452(41.94)	1917
S	0.195 (21.27)	0.107 (12.81)	0.056 (8.08)	0.08 (10.94)	0.563 (46.89)	678
GI	0.239 (24.03)	0.187 (19.8)	0.163 (18.76)	0.158 (17.59)	0.253 (19.83)	262

**Table 14:** The probability of failure of pipe materials given the closeness of the road (within 25 m) $P\left({M}_{i}|{F}_{l}\right)$.

	CI	PE	DI	S	GI	${l}_{r}$
0–5	0.067 (11.24)	0.288 (17.48)	0.203 (48.83)	0.083 (19.3)	0.359 (3.16)	1647
5–10	0.027 (7.42)	0.393 (29.4)	0.143 (43.44)	0.034 (13.74)	0.403 (6)	1050
10–15	0.022 (6.96)	0.324 (28.16)	0.116 (43.92)	0.03 (13.12)	0.507 (7.84)	662
15–20	0.02 (6.76)	0.296 (30.06)	0.092 (39.81)	0.03 (14.42)	0.562 (8.96)	515
20–25	0.019 (6.28)	0.28 (34.05)	0.071 (36.3)	0.022 (12.32)	0.608 (11.05)	445

**Table 15:** The probability of failure of pipes experience traffic ($\times {10}^{3}$ AADT) on the road within 25 m given pipe materials $P\left({F}_{l}|{M}_{i}\right)$.

	0–8	8–16	16–25	25–36	36–51	> 51	${l}_{r}$
CI	0.227 (21.29)	0.339 (31.35)	0.192 (19.29)	0.133 (14.52)	0.046 (5.78)	0.063 (7.78)	369
PE	0.236 (24.38)	0.318 (31.25)	0.175 (16.48)	0.113 (13.2)	0.061 (5.25)	0.098 (9.44)	1076
DI	0.214 (24.15)	0.326 (28.65)	0.184 (17.25)	0.136 (13.37)	0.054 (4.58)	0.086 (12)	1882
S	0.182 (20.72)	0.283 (26.7)	0.206 (18)	0.164 (15.19)	0.06 (6.08)	0.104 (13.31)	666
GI	0.216 (23.34)	0.348 (32.45)	0.17 (17.68)	0.137 (11.92)	0.041 (5.05)	0.088 (9.56)	261

**Table 16:** The probability of failure of pipe materials given the traffic ($\times {10}^{3}$ AADT) on the road within 25 m given $P\left({M}_{i}|{F}_{l}\right)$.

	CI	PE	DI	S	GI	${l}_{r}$
0–8	0.038 (7.9)	0.344 (26.38)	0.138 (45.71)	0.039 (13.88)	0.442 (6.13)	2553
8–16	0.038 (9.22)	0.309 (26.82)	0.14 (43.02)	0.04 (14.18)	0.474 (6.76)	2998
16–25	0.04 (9.63)	0.32 (23.99)	0.149 (43.93)	0.055 (16.21)	0.435 (6.25)	2049
25–36	0.038 (9.24)	0.281 (24.51)	0.149 (43.42)	0.059 (17.45)	0.473 (5.38)	563
36–51	0.033 (9.8)	0.391 (25.94)	0.152 (39.6)	0.056 (18.6)	0.367 (6.07)	1008
> 51	0.026 (6.11)	0.348 (21.62)	0.134 (48.07)	0.054 (18.88)	0.438 (5.32)	717

**Table 17:** The probability of failure of pipes within 100 m to railway infrastructure given pipe materials $P\left({F}_{l}|{M}_{i}\right)$.

	0–20	20–40	40–60	60–80	80–100	${l}_{r}$
CI	0.288 (15.68)	0.184 (18.73)	0.181 (34.05)	0.194 (14.75)	0.153 (16.78)	145
PE	0.201 (17.36)	0.289 (25.36)	0.167 (20.91)	0.181 (18.81)	0.162 (17.55)	486
DI	0.299 (16.95)	0.209 (22.17)	0.18 (28.92)	0.153 (15.44)	0.16 (16.5)	625
S	0.343(17.17)	0.311 (24.31)	0.122 (33.2)	0.109 (12)	0.114 (13.32)	313
GI	0.244 (14.46)	0.268 (24.89)	0.133 (23.61)	0.144 (20.73)	0.211 (16.28)	138

**Table 18:** The probability of failure of pipe materials given the proximity of railway infrastructure (within 100 m) $P\left({M}_{i}|{F}_{l}\right)$.

	CI	PE	DI	S	GI	${l}_{r}$
0–20	0.039 (10.57)	0.279 (21.69)	0.142 (38.63)	0.059 (22.17)	0.481 (6.94)	468
20–40	0.023 (6.81)	0.362 (30.85)	0.09 (34.72)	0.048 (19.04)	0.478 (8.58)	399
40–60	0.039 (7.95)	0.37 (29.4)	0.137 (36.98)	0.033 (18.73)	0.42 (6.94)	287
60–80	0.041 (8.81)	0.385 (30.79)	0.112 (37.27)	0.029 (15.05)	0.434 (8.09)	277
80–100	0.028 (7.78)	0.296 (33.17)	0.101 (35.07)	0.026 (13.62)	0.55 (10.36)	275

**Table 19:** The probability of failure of pipes being operated at various pressure ranges (psi) given pipe material $P({F}_{l} |{M}_{i} )$.

	18–54	54–83	83–106	106–134	134–168	> 168	${l}_{r}$
CI	0.067 (9.34)	0.288 (22.33)	0.3 (32.19)	0.246 (26.66)	0.079 (7.85)	0.02 (1.63)	464
PE	0.071 (9.02)	0.299 (29.33)	0.37 (37.94)	0.155 (15.03)	0.093 (6.52)	0.012 (2.15)	2691
DI	0.072 (11.82)	0.258 (29.68)	0.308 (33.85)	0.169 (13.05)	0.167 (7.86)	0.026 (3.75)	3055
S	0.14 (39.61)	0.297 (13.6)	0.286 (32.39)	0.14 (9.1)	0.094 (3.71)	0.044 (1.59)	2098
GI	0.106 (9.75)	0.299 (33.53)	0.336 (33.88)	0.14 (13.38)	0.076 (6.44)	0.042 (3.01)	1076

**Table 20:** The probability of failure of pipe material given various pressure ranges (psi) $P({M}_{i} |{F}_{l} )$*.*

	CI	PE	DI	S	GI	${l}_{r}$
18–54	0.012 (2.74)	0.178 (15.33)	0.053 (22.81)	0.039 (52.49)	0.718 (6.62)	1583
54–83	0.017 (4.24)	0.243 (32.27)	0.061 (37.08)	0.027 (11.67)	0.652 (14.75)	2446
83–106	0.016 (4.6)	0.261 (31.42)	0.064 (31.84)	0.022 (20.92)	0.638 (11.22)	3248
106–134	0.029 (9.8)	0.252 (32.06)	0.081(31.59)	0.025 (15.14)	0.613 (11.41)	1262
134–168	0.016 (6.08)	0.256 (29.3)	0.135(40.07)	0.029 (12.98)	0.565 (11.57)	599
> 168	0.01 (3.07)	0.085 (23.58)	0.054(46.61)	0.034 (13.55)	0.816 (13.2)	246

**Table 21:** The probability of failure of pipes distributing different types of water given pipe material $P({F}_{l} |{M}_{i} )$.

	FW	SW	${l}_{r}$
CI	0.467 (51.87)	0.533 (48.13)	491
PE	0.484 (73.52)	0.516 (26.48)	2843
DI	0.408 (72.25)	0.592 (27.75)	3293
S	0.72 (91.39)	0.28 (8.61)	2200
GI	0.998 (99.65)	0.002 (0.35)	1117

**Table 22:** The probability of failure of pipe material given various types of distributing water $P\left({M}_{i}|{F}_{l}\right)$.

	CI	PE	DI	S	GI	${l}_{r}$
FW	0.01 (3.24)	0.147 (26.63)	0.038 (30.32)	0.024 (25.62)	0.782 (14.19)	7847
SW	0.048 (11.27)	0.673 (35.91)	0.234 (43.6)	0.04(9.04)	0.006 (0.19)	2096

$({l}_{r})$, with the percentage of the cumulative length depicted in parentheses for each cell. It should be noted that for the probability of a certain factor given a material, the sum of the rows for each material will be 1. Conversely, for the probability of failure of a certain pipe material given a certain factor, the sum of the rows will be 1 for each factor range. The reason for this is rooted in the nature of conditional probabilities. When calculating the probability of a factor being given a material, we consider all possible factor outcomes for that specific material. These outcomes must collectively account for 100% of the possibilities, hence summing to 1.

Similarly, when calculating the probability of a pipe material failing given a certain factor, we consider all possible material outcomes for that specific factor range. Again, these outcomes must represent all possibilities, summing to 1. This summing pattern is a direct result of the axioms of probability, specifically that the sum of probabilities for all possible outcomes in a given scenario must equal 1. In this study, each row represents a complete set of possible outcomes for a given condition (either a specific material or a specific factor range), thus adhering to this fundamental principle of probability theory.

### Pipe-related factors

#### Age

Table 1 illustrates the trends of failure probability after a certain duration for different pipe materials, while Table 2 presents the probability of failure of different pipe materials given their age. In Table 1, the rows represent the different materials, and the columns represent various age ranges. The values in the table are interpreted as the probability of failure. CI pipes show a low probability of failure at an early age, and a higher probability of failures can be expected at the end of their service life. The increasing trend of failure probabilities of CI pipes can be associated with corrosion, as damage due to corrosion increases with time. This trend agrees with previous studies^36–38^. DI shows the highest failure probability within 15–27 years of installation, an observation that tallies with the result reported by Singh^19^.

The probability of experiencing a failure for PE, GI, and S pipes was found to be highest within the first 15 years of their installation. This trend gradually decreases in the later part of the pipes’ age. One of the reasons for the high failure probability of the pipes at an early age may be due to installation and manufacturing flaws. This justification is well established in the extant literature^5,11^. According to Yang et al.^39^, an inefficient tapping process during the installation of PE pipes is one of the problems that can cause catastrophic failure at an early stage. Yang et al.^39^ reported a burnt mark on a failed PVC pipe hole attributed to high temperature during tapping. This poses a significant issue since PE pipes cannot dissipate heat quickly when exposed to high temperatures. Therefore, it is essential to ensure proper tapping processes when installing plastic pipes to prevent catastrophic failures. Plastic pipes located in Honolulu WDN were also found to exhibit the highest failure probabilities within 20 years of installation^19^.

Table 2 presents the failure probability for various pipe materials based on their age, which aligns with the findings in Table 1. The first row suggests that GI and PE pipes are more likely to fail during their early stages in a WDN than other materials. Except for the last row, all other rows indicate that GI pipes have a higher probability of failure than any other material. Additionally, CI pipes are expected to have more failures after 62 years of service life.

#### Diameter

The pipes are divided into two groups based on their diameter distribution. Group A consists of GI and PE pipes with diameters ranging from 20 to 175 mm, while Group B consists of CI, DI, and S pipes with diameters ranging from 20 to 500 mm. Given the material types, the probability of failure due to pipe diameter is presented in Tables 3 and 5. Generally, the probability of failure decreases in GI, PE, CI, and DI pipes as the diameter increases. This finding is consistent with previous studies and may be attributed to several factors^5,40,41^. Firstly, small-diameter pipes exhibit thinner wall thickness, making them more susceptible to damage from external factors such as ground and soil movements or nearby construction activities.

Further, small-diameter pipes are more prone to clogging and blockages, potentially increasing the pressure and stress on the pipes causing their ultimate failure^12^. Conversely, S pipes exhibit a higher probability of failure in larger diameters. This is primarily because these pipes are often used as water mains, transporting substantial volumes of water and enduring higher pressures, leading to increased structural stress and potential failure. Additionally, large-diameter pipes of this type tend to be older and more prone to corrosion and other forms of deterioration, further elevating the likelihood of failure^11^.

Tables 4 and 6 show the probability of failure based on material types for given pipe diameters. The results indicate that GI and DI pipes exhibit the highest failure probability regardless of diameter, except for pipes exceeding diameters of 150 mm and 450 mm, respectively. This suggests that GI and DI pipes may need more frequent inspections and maintenance to reduce the risk of failures.

### Environmental factors

Tables 7, 8, 9, 10, 11, 12, 13, 14, 15, 16, 17, 18 present information regarding the environment-related factors of HK’s WDN, including factors such as land use (urban, rural, waterbody, and sea), soil resistivity, different geographical locations, and the effect of dynamic loading in HK.

#### Land use

Table 7 shows the probability of failure due to land use given pipe material. The results typify that urban areas have higher failure probabilities for most pipe materials, with CI, PE, DI, S, and GI having probabilities of 0.96, 0.73, 0.90, 0.81, and 0.48, respectively. The high density of civil and electrical infrastructure in urban areas may contribute to these failures, possibly due to dynamic loading from traffic, stray current from electrical infrastructure, or malpractices during construction or repair work^42^. GI pipes show similar failure probabilities in both rural and urban areas.

The failure probability of the material types given land use is shown in Table 8. According to the results, GI and PE have the highest failure probabilities in the urban and rural areas of HK. At the same time, GI and S pipes exhibited the highest failure probabilities for pipes located in waterbody and sea. This can be attributed to the higher chances of corrosion in metallic pipes used in wet areas. Exposure to elements such as saltwater can exacerbate the corrosion process, leading to higher failure probabilities^43^.

#### Soil corrosivity

The failure probability of the pipes due to soil corrosivity, given their material types, is reported in Table 9. A closer look at the table indicates that pipes in mildly-corrosive soil exhibited the highest failure probability. Although one would have expected that pipes in highly-corrosive soil may experience the most failures^44^, it is essential to note that mildly-corrosive soil may cause gradual and prolonged damage to pipes, leading to an increased probability of failure over time. Conversely, highly corrosive soil may cause rapid and severe damage to pipes, resulting in early failures that are quickly detected and repaired. Therefore, the results highlight the need for regular monitoring and maintenance of pipes located in all types of corrosive soil to prevent failures and ensure the longevity of the water distribution network. WSD HK uses Linear Polarization Resistance (LPR) as a metric to define the corrosivity of the soil.

Moreover, the probability of failure of pipe materials, given their soil corrosivity, is indicated in Table 10. The results show that GI and PE have the highest failure probabilities for highly and mildly corrosive soil pipes. This suggests that the susceptibility to corrosion is a significant factor affecting the performance of GI and PE pipes, regardless of the level of soil corrosivity. This is consistent with previous studies showing that corrosion is a significant cause of failure in metal pipes^12,45, 46^. The higher failure probabilities in highly-corrosive soil can be attributed to the accelerated corrosion rate caused by the corrosive soil environment. Although PE is a plastic pipe that is typically resistant to corrosion^5^, it can still be impacted by environmental factors such as UV radiation, high temperatures, and chemical exposure, leading to the formation of weak spots that are more susceptible to corrosion.

#### Reclaimed area

Hong Kong’s surface area keeps increasing due to several reclamation projects (Development Bureau and Construction Industry^26^). Most of the reclaimed areas were occupied by sea before the reclamation. Table 11 shows the failure probabilities of pipes due to reclamation given their pipe materials, while Table 12 presents the failure probability due to the pipe materials given their reclamation status. N and Y represent pipes in non-reclaimed and reclaimed areas, respectively. DI, CI, and S pipes were the most affected, as they exhibit failure probability of 0.37, 0.33, and 0.27, respectively. The higher failure in the sea-reclaimed land associated with these pipes can be attributed to the higher susceptibility of these materials to external stresses and movements, such as settlement and ground deformation, common in reclaimed areas^47^. These stresses can cause damage to the pipes, such as cracks or leaks, which increases the likelihood of failure. Additionally, the changes in soil characteristics and composition in reclaimed areas can lead to increased corrosion rates, further contributing to the higher failure probabilities of these materials in such areas^48^. Water authorities need to consider these factors when selecting materials and designing pipelines in reclaimed areas to ensure the long-term reliability and safety of the water distribution network.

#### Distance from the road

Hong Kong is known for having one of the highest population densities globally, resulting in a highly concentrated urban infrastructure network to meet the population’s needs^49,50^. This network comprises surface and buried infrastructures, including water pipelines, gas pipelines, power cables, roads, underground/surface transit railways, and traction power lines. Due to vehicular traffic and transit railway networks, the buried pipelines experience continuous dynamic loading and unloading cycles.

Table 13 presents the failure probability of the pipes located within 25 $m$ from the road, given the pipe materials. The result demonstrates that brittle pipes like S and CI have a higher probability of failure if they are within a 10-m distance of roads. The probability of failure decreases as the distance between the pipes and roads increases. On the other hand, materials like DI and PE are comparatively flexible and exhibit no significant increase or decrease in the failure probability across various distance ranges, except for the last row (20–25 $m$). The failure probability for different pipe materials given various ranges of the distance between roads and water pipes is reported in Table 14. According to the result, GI and PE pipes do not seem to be affected by the distance from the road as their failure probabilities increase as the pipes get farther from the road, showing that other factors may have influenced the higher failure probabilities.

#### Traffic

The location of buried pipes is critical in determining their susceptibility to failure. In some cases, pipes are buried directly above highways, raising concerns about vehicular traffic’s impact on their integrity. The effect of annual average daily traffic (AADT) on pipe failure probability was examined to investigate this issue. The probability of failure due to AADT given the pipe materials and the failure probability of the various pipe materials given the AADT are recorded in Table 15 and Table 16, respectively. The results indicate that all the pipe materials experienced the highest failure probability when subjected to AADT of about 8–16 $\times {10}^{3}$ AADT suggests that vehicular traffic intensity significantly impacts the pipes’ structural integrity^42^. AADT is known to cause dynamic loading and unloading on buried pipelines, which can result in cyclic stresses that may weaken the pipes over time^5^. Moreover, the magnitude and frequency of traffic-induced loads can be influenced by vehicle speed, axle load, and pavement conditions^51^.

#### Distance from mass transit railway

Hong Kong’s comprehensive mass transit railway (MTR) system is a crucial transportation network, connecting most population centers through surface and subway trains. The railway system employs three primary power contact lines, namely 550 V DC, 1.5 kV DC, and 25 kV AC. This section investigates the influence of the MTR system on pipe failure.

Table 17 shows the probability of failure when the pipe is located at certain distances from the MTR system, given the pipe materials. In contrast, Table 18 represents the failure probability of the different pipe materials, given their proximity to the MTR system. Generally, the result shows that the proximity of the MTR system affects the pipe failure for metallic pipes such as CI, DI, and GI. The increased failure probability when the MTR system is close to the pipe’s location can be attributed to several reasons. First, the construction and maintenance of the railway infrastructure, such as tunnels and underground stations, can cause ground settlement and soil displacement, which can, in turn, damage and even rupture buried pipes. Second, the operation of trains, particularly heavy freight trains, can cause ground vibration and dynamic loading on buried pipes, leading to fatigue failure over time^11^. Third, the traction power lines that supply power to the trains can create electric and magnetic fields that may induce stray currents in nearby buried pipes, leading to corrosion and eventual failure^52^.

### Operation-related factors

Based on the framework developed in this study, internal water pressure and water type are the two significant factors influencing water pipe failure in HK WDN.

#### Internal water pressure

The failure probability due to internal water pressure given the pipe materials is reported in Table 19. It reveals that the probability of failure increases for all pipe materials as the internal water pressure rises. This trend continues until the pressure reaches a range of 83–106 psi, where the probability of failure decreases. While this shows that an increase in pressure increases the likelihood of failure to a certain point, the decrease in failure probability of the pipes beyond 106 psi may be attributed to some reasons. It is possible that the pressure range is not uniformly distributed, and certain sections of the pipes are subjected to higher pressures than others. In such cases, the failure probability may increase in those sections where the pressure is highest but decrease in other sections where the pressure is lower^5,10^. It is also important to note that the decrease in failure probability may not be linear and that other factors may be at play that influence the behavior of the pipes under different pressure levels. Table 20 presents the pipe materials’ failure probability given the water pressure. As seen in Table 20, GI and PE pipes are more susceptible to failure than other pipes in the WDN. This highlights the need for proper material selection for pipes likely to experience higher water pressure levels.

#### Water type

The pipes in HK’s WDN either carry freshwater or saltwater. This section investigates the influence of water type on the probability of pipes’ failure. The probability of failure due to water type given the pipe materials is provided in Table 21, which denotes that the probability of failure in saltwater-carrying pipes is higher than that of freshwater pipes. This could be attributed to several factors, such as the chemical composition of salt water, which may cause corrosion or other forms of degradation in the pipes over time^5^. Moreover, saltwater pipes may be exposed to more extreme environmental conditions, such as tidal movements or high levels of sunlight, which can further contribute to their deterioration and subsequent failure^53^.

On the other hand, Table 22 indicates the failure probability of the different types of pipe material, given that their water type shows GI to be more susceptible to failure than other pipe materials, followed by PE for freshwater pipes. However, PE exhibited the highest failure probability for saltwater. Saltwater can cause PE pipes to swell and absorb water, reducing the pipe’s structural integrity. Additionally, saltwater can cause the material to become brittle over time, leading to cracks and fractures that increase the likelihood of failure^54^.

## Theoretical and practical implications

### Theoretical implications

The proposed framework for analyzing the probability of failure of water pipes in a WDN has significant theoretical implications. By selecting the critical factors affecting failure through two different algorithms, one for numerical and one for categorical data, the framework provides a more comprehensive understanding of the factors influencing water pipe failure. The algorithms utilized in the framework ensure precise and accurate identification of the critical factors, thereby enhancing the reliability and validity of the results obtained. Further, the framework employs Bayes’ theorem and the likelihood feature method to determine the conditional failure probabilities of pipes, addressing the bias associated with historical failure data. This approach can be applied to any WDN or asset experiencing failure, regardless of size and complexity, making it a valuable tool for researchers and practitioners in water infrastructure management.

### Practical implications

The proposed framework offers significant practical implications for managing and maintaining WDNs. At its core, this approach enables a more nuanced and accurate assessment of pipe failure probabilities, which in turn facilitates more informed decision-making processes across various aspects of WDN management.

One of the primary advantages of this framework is its ability to identify and prioritize critical factors influencing water pipe failures. Water utility managers can adopt a more targeted and efficient maintenance strategy by pinpointing these key determinants. This focused approach allows for the optimal allocation of often limited resources, potentially leading to substantial cost savings while simultaneously enhancing the overall reliability of the network. The framework’s capacity to provide a more reliable assessment of pipe failure probabilities is particularly valuable for asset management. Armed with this refined information, decision-makers can make more informed choices regarding pipe replacement or rehabilitation. This enhanced predictive capability allows for prioritizing high-risk assets, ensuring that maintenance efforts are directed where they are most needed. Consequently, this approach can significantly reduce the risk of unexpected failures, minimizing service disruptions and associated costs.

Moreover, the probabilistic nature of this framework aligns well with risk-based asset management strategies. It provides a quantitative basis for risk assessment, allowing managers to balance the likelihood of failure against the potential consequences. This risk-based approach can be instrumental in justifying budget allocations and investment decisions, as it provides a clear, data-driven rationale for prioritizing certain assets or areas of the network over others. The framework also holds potential for long-term planning and scenario analysis. By understanding how different factors contribute to failure probabilities, managers can model various future scenarios, such as the effects of climate change or urban development on their networks. This forward-looking capability can inform long-term infrastructure planning and investment strategies, helping utilities to build more resilient and sustainable networks.

## Conclusion

The negative impacts of water pipe failure are numerous, including flooding, disruption of business and services, erosion, increased maintenance, and rehabilitation costs. The factors affecting the failure of the pipes are often categorized into pipe-related, environment-related, and operation-related factors. Therefore, it is necessary to mitigate the failure of these pipes by analyzing the historical failure data of a WDN to estimate the failure probability of each pipe material and the factors influencing the failure. The existing literature is limited by the absence of a systematic selection of significant factors affecting water pipes and addressing the bias associated with the frequency distribution of assets in a WDN. Hence, this study aims to fill these gaps by proposing a new framework that systematically identifies the most significant factors influencing the failure of pipes in a particular WDN. The framework is composed of two algorithms, with each algorithm consisting of three statistical tests. One algorithm is meant for numerical data, while the other is developed for categorical data.

Applying the proposed framework to HK WDN shows that 10 out of 21 factors influencing pipe failure are significant. For the pipe-related factors, it was generally found that the failure probability of the pipes increases with age, except for brittle pipes such as PE, which experience a higher failure probability within 15 years of installation. The results also indicate that smaller-diameter pipes are prone to more failure than larger ones. The significant environment-related factors include land use, soil corrosivity, reclaimed area, distance from the road, traffic, and distance from mass transit railway, and their associated justification for influencing pipe failure has been discussed. Concerning the operation-related factors, failure probability was found to increase generally with internal pressure, provided all other conditions remain constant. The result also typifies that higher failure probabilities are associated with saltwater pipes than freshwater pipes. Regarding the pipe materials, GI and PE were the most vulnerable pipes in the network.

Although the results of this analysis are directly applicable to the case study, the framework developed in this study can be applied to any other WDN. By utilizing the proposed framework, decision-makers in the water infrastructure sector can prioritize the most influential failure factors in their networks and allocate resources effectively, thereby reducing the risk of failures and minimizing the associated economic, environmental, and social consequences. While the framework developed in this study contributes to the field of water pipe failure analysis, it is noted that future research can be conducted to explore integrating the framework into existing asset management systems to provide a more holistic approach to water pipe management.

## Data Availability

The data used in this study is confidential and cannot be shared with the public due to the confidentiality agreement between the authors and the funding agency. This agreement ensures that the data remains confidential and is only used for the purpose of the research project. As such, the authors do not have the right to share the data with the public. We have taken steps to ensure that the methods used in this study are clear and replicable.
